# Supplementary material for: Dopamine release in human associative striatum during reversal learning
Source: Nat Commun. 2024 Jan 2;15:59. doi: 10.1038/s41467-023-44358-w (PMC10762220; doi:10.1038/s41467-023-44358-w)
Supplement: Supplementary file 1 — Supplementary Information [file 41467_2023_44358_MOESM1_ESM.pdf]

**Supplementary material to: Dopamine release in human associative striatum during reversal learning**

Contains:

- Dopamine-behavior association based on other models: **Supplementary Figure 1**
- Behavioral associations with static binding potentials and choice of window size: **Supplementary Figure 2** plus discussion
- Behavioral model fitting: **Supplementary tables 1 and 2**, **Supplementary Figure 3**, plus discussion
- PET control analyses: Simulations, additional fit*s* and single subject data supplementary: **Supplementary Figures 4, 5, and 6** plus discussion.
- FMRI model information: **Supplementary Figure 7** plus discussion
- BOLD-PET correlations: **Supplementary Figure 8** plus discussion

## Dopamine-behavior associations based on other models

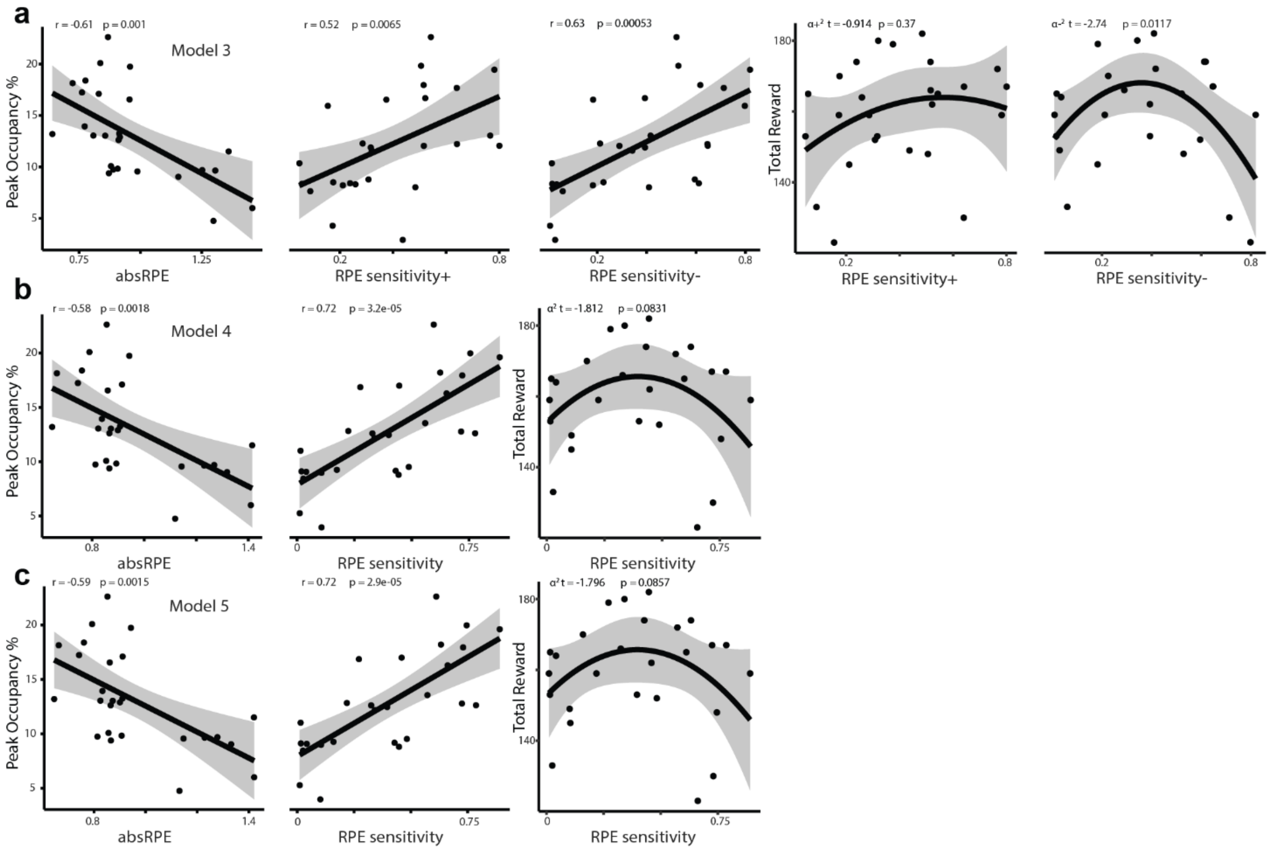


**Fig. 1.**

**Behavioral associations with other models and less conservative DA release cluster.** All linear associations are Pearson correlations, all non-linear associations are linear regression models with RPE sensitivity and its quadratic term added as predictors. All associations with N = 26 participants. (**a**) Model 3 had separate RPE sensitivity estimations depending on if the outcome was rewarded (+) or not (-). Using this model the association with absRPE and peak occupancy was similar to Model 1. Both RPE sensitivity + and – was associated with peak occupancy. Only RPE sensitivity to non-rewarded outcomes showed a nonlinear association with total reward. (**b**) Model 4 dynamically updated RPE sensitivity depending on choice confidence. This model yielded the same conclusions as Model 1. (**c**) Model 5 was identical to Model 4 but with different values for fixed variables. This model yielded the same conclusions as Model 1. The peak occupancy for these correlations were extracted from a less conservative cluster than in the main manuscript, indicating that the behavioral correlations are robust to both model selection and the specific threshold of the DA release cluster. Shaded areas represent 95% confidence interval. Source data are provided as a Source Data file. absRPE – absolute Rward Prediction Error, RPE – Reward Prediction Error.

## Behavioral associations with static binding potentials and choice of window size

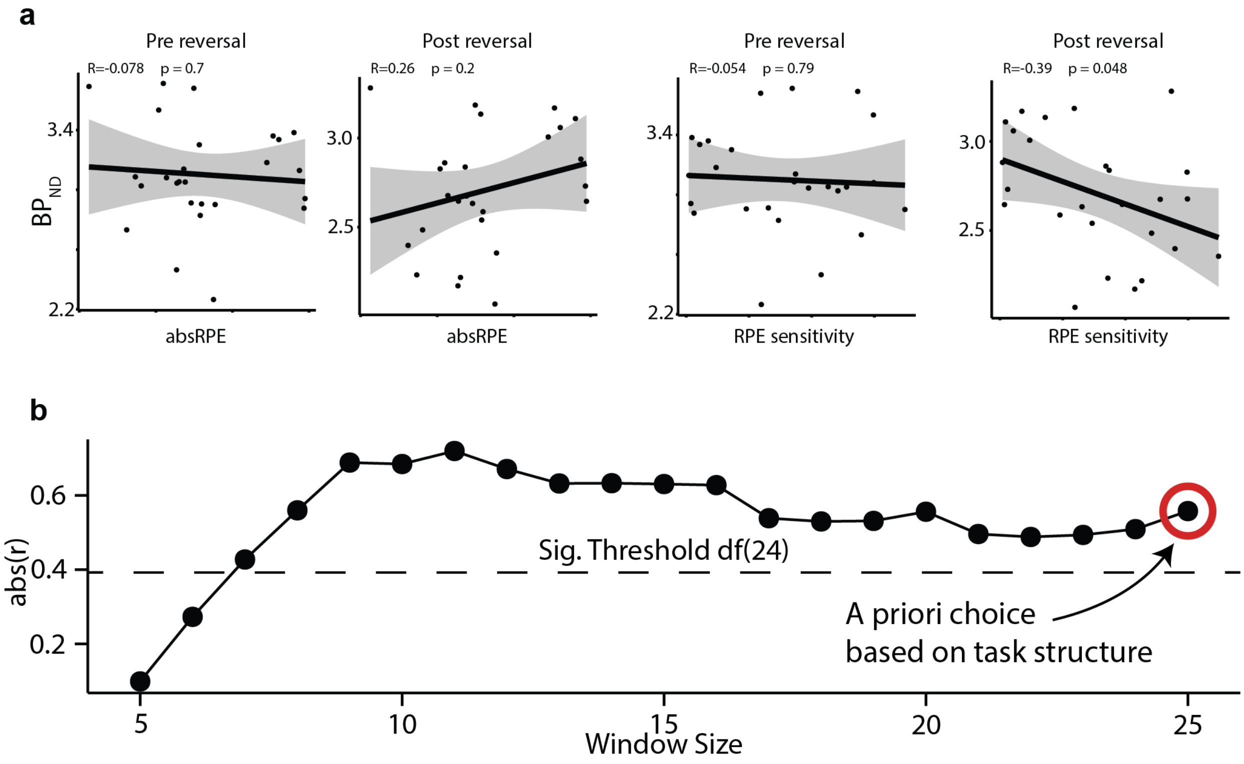


**Fig. 2.**
**Behavioral associations with static binding potentials and choice of window size.** (**a**) Associations between absRPE, RPE sensitivity and static $BP_{ND}$ as estimated from pre and post reversal. Only the association between RPE sensitivity and post reversal $BP_{ND}$ reached significance ($p = 0.047$), no other significant associations ($p > 0.05$) was observed between the static $BP_{ND}$ and absRPE or sensitivity. This control analysis suggest that there is important information in the difference between pre and post reversal $BP_{ND}$. All linear associations are Pearson correlations. All associations with $N = 26$ participants. Shaded areas represent 95% confidence interval. (**b**) Different window sizes to calculate the mean absRPE for Pearson correlation with occupancy. This control analysis show that the obtained result between absRPE and occupancy is robust for window size 7 to 25. The a priori choice of window size 25 was chosen due to how the task paradigm was designed (i.e. all trials within the first reversal block). Source data are provided as a Source Data file. absRPE – Absolute Reward Prediction Error, RPE – Reward Prediction Error, $BP_{ND}$ – Binding Potential, Sig. – Significant.

To make sure that the observed behavioral associations were not driven by the static binding potentials estimated from the stable task period (pre reversal) nor the volatile task period (post reversal) the key correlations (absRPE and RPE sensitivity) in the main manuscript (see Main Manuscript; Fig. 2d) were correlated with these binding potentials using Bayes factor correlation (null hypothesis tested against an alternative hypothesis of a medium correlation of $r = 0.33$). Bayes factor for pre reversal binding potential associated with absRPE was 0.45, for post reversal binding potential associated with absRPE was 0.84. Bayes factor for pre reversal binding potential associated with RPE sensitivity was 0.43, for post reversal binding potential associated with RPE sensitivity was 2.18. These Bayes factors generally indicate in favor for the null hypothesis. In contrast, the dopamine occupancy association with absRPE and sensitivity had Bayes factors of 15.76 and 25.30 respectively, indicating strong evidence in favor for the alternative hypothesis (Fig. 2a). This analysis highlights that the difference in binding potential (i.e. amount of DA release) is driving the associations. A key finding was that DA occupancy was significantly associated with mean absRPE over the trials corresponding to the first reversal (window size of 25 trials). To make sure that this association was not biased to the a priori defined window size, we performed the same

correlation with window sizes of 5 to 25 trials. At window size 7, the association became significant and remained significant for larger window sizes (Fig. 2b).

## Behavioral model fitting

The behavioral data were fit using hierarchical models with hyperpriors for each group-level free parameter's mean and variance. The hyperpriors were weakly informed using a normal distribution with a mean of 0 and standard deviation of 1. The individual level free parameters were given the same priors. STAN in R4.1.1 was used to fit the data with 4 chains, 6000 iterations (5000 warmup), yielding 4000 posterior estimates for each parameter and individual, the mean of the posterior estimates were used in the parameter recovery analysis and the correlation analysis (RPE sensitivity (α) and peak dopamine occupancy; Main Manuscript Fig. 2d).

Five models from prior work were selected as candidate computation models of behavior, with the main goal to extract trial-wise RPE and RPE sensitivity estimates. First, a basic Rescorla-Wagner reinforcement learning model[1,2] was assessed (Model 1). Model 1 included an equation for choice probability using a softmax rule:

$$P(A)_t = \frac{\exp(\beta \times V(A)_t)}{\exp(\beta \times V(A)_t) + \exp(\beta \times V(B)_t)} \quad (1)$$

Where $P(A)_t$ is the probability of choosing the index finger at trial $t$, $V(A)_t$ is the value of the index finger choice at time $t$, $V(B)_t$ is the value of the middle finger choice at time $t$, and $\beta \in [0,\text{Inf}]$ is the inverse temperature to be estimated from the data. Value updating of the index and middle finger choice was calculated from the equations:

$$V(A)_{t+1} = V(A)_t + \alpha(R_t - V(A)_t) \quad (2)$$

Where $R_t$ is the reward (0 or 1) at time $t$, and $\alpha \in [0,1]$ is the RPE sensitivity (often referred to as learning rate) to be estimated from the data. Since all participants were trained on a task version where there was a 50% chance of reward for either choice, the initial value of the two choices were set to 0.5.

As prior work has suggested to fit $\alpha$ separately when learning contexts differ in terms of their stability[38] , Model 2 extends on Model 1 by fitting an identical model but with separate $\alpha$ for the different task periods ($\alpha_1$, $\alpha_2$, $\beta$). Next, because previous studies have shown that RPE sensitivity can be different depending on the valence of the outcome [3,4], Model 3 included separate estimations of $\alpha$ depending on the valence of the outcome:

$$\begin{aligned} V(A)_{t+1} &= V(A)_t + \alpha^{+}(R_t - V(A)_t), R = 1 \\ V(A)_{t+1} &= V(A)_t + \alpha^{-}(R_t - V(A)_t), R = 0 \end{aligned} \quad (3)$$

Previous work has also argued that RPE sensitivity depends on the average reward rate[5–7]. In the current experiment, it is conceivable that the average reward rate is being manipulated during the transition from stable to volatile period as well as during each reversal. We therefore fitted a model that included a meta-cognitive layer of parameters that dynamically controlled the lower level RPE sensitivity parameter (Model 4). The model included a choice confidence estimation[8]:

$$\begin{aligned} C_{t+1} &= C_t + \gamma \times \frac{(2-|\delta_t|)}{(2-C_t)} \quad (4) \\ \delta &= R_t - V(A)_t \end{aligned}$$

where $\gamma$ is confidence sensitivity and $\delta$ is the RPE. RPE sensitivity was then updated according to:

$$\alpha = \frac{(\alpha_0 + \kappa \times C_t)}{(1+\kappa \times C_t)}, R = 1 \quad (5)$$

$$\alpha = \frac{\alpha_0}{(1+\kappa\times C_t)}, R = 0 \qquad (6)$$

where $\alpha_0 \in [0,1]$ is a free parameter representing RPE sensitivity when confidence is zero (i.e. the baseline RPE sensitivity that that $\alpha$ was modified against depending on moment to moment choice confidence) and $\kappa \in [0,1]$ is a free parameter used as weight on confidence. As confidence increases $\alpha$ approaches 1 for rewarding outcomes while it approaches 0 for non-rewarding outcomes. In a constant but noisy environment, the effect of confidence is to stabilize a representation of the current learned reward contingencies by suppressing sensitivity to noise. A volatile environment decreases confidence, causing sensitivity to contradictory outcomes to increase and sensitivity to confirmatory outcomes to decrease until a new set of reward contingencies is learned. Because a first-pass estimation of Model 4 showed that it was unstable, the number of estimated parameters was reduced so that $C_0$ (i.e. initial confidence) as well as $\gamma$ was fixed to 0.5 (Model 4) or 0.1 (Model 5).

The models were compared using maximum likelihood estimates as well as AIC and BIC to assess which model best accounted for the data. A parameter and model recovery analysis was performed on three candidate models. In short, synthetic data was created from the mean and covariance of the estimated parameters under the real data. The synthetic data was then fitted using the same model that generated it (parameter recovery) as well as with the other models (model recovery). All models were fitted using STAN in R4.1.1.

***Recovery analysis and evaluation of behavioral models***

**Table 1. Model comparison.**

| Model # | Estimated Parameters | Fixed Parameters | Likelihood | AIC | BIC |
|---|---|---|---|---|---|
| 1 | $\alpha$, $\beta$ | - | -98.75 | 201.49 | **204.01** |
| 2 | $\alpha_1$, $\alpha_2$, $\beta_1$ | - | -114.28 | 234.57 | 238.34 |
| 3 | $\alpha+$, $\alpha-$, $\beta$ | - | **-97.61** | **201.22** | 204.99 |
| 4 | $\alpha_0$, $\beta$, $\kappa$ | $C_0 = 0.5$, $\gamma = 0.5$ | -98.92 | 203.84 | 207.67 |
| 5 | $\alpha_0$, $\beta$, $\kappa$ | $C_0 = 0.5$, $\gamma = 0.1$ | -98.90 | 203.81 | 207.58 |

Likelihood estimation of model space and each computational cognitive model's associated parameters. Bold indicates best value. AIC - Akaike information criterion, BIC – Bayesian information.

**Table 2. Parameter recovery.**

| Model # | Parameters | | |
|---|---|---|---|
| 1 | α | - | β |
| | 0.74 | - | 0.70 |
| 3 | α+ | α- | β |
| | 0.74 | 0.82 | 0.84 |
| 4 | $\alpha_0$ | κ | β |
| | 0.73 | 0.12 | 0.91 |
| 5 | $\alpha_0$ | κ | β |
| | 0.74 | 0.11 | 0.90 |

Parameter recovery statistics. Values under each parameter represent Pearson's correlation coefficients.

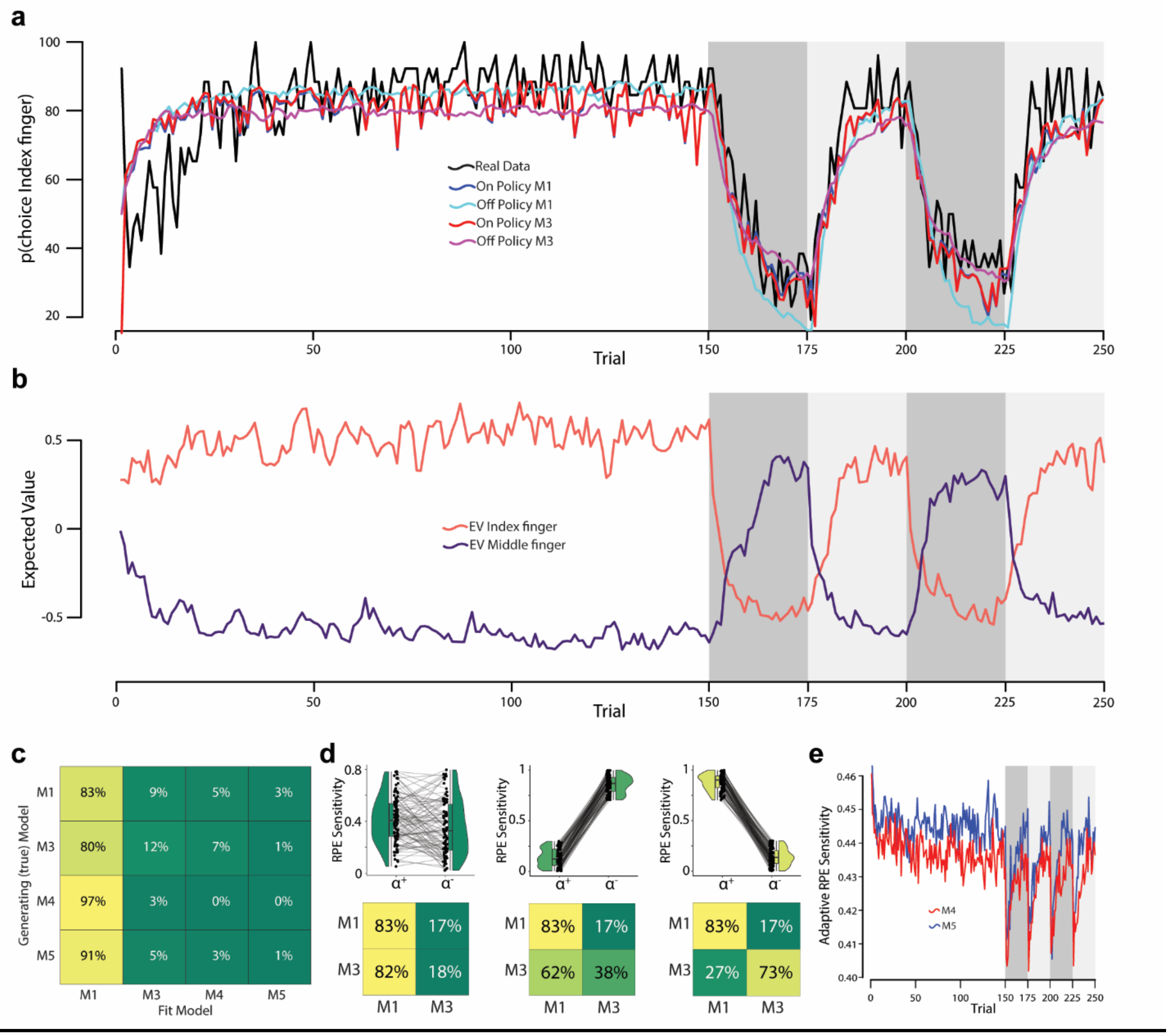


**Fig. 3.**

**Choice prediction and expected value.** (**a**) Real (N = 26 participants), on policy, and off policy choice probability (N = 100 simulations). On policy choice probability reflect model choice based on the parameters that were fit to the real data. Off policy choice probability reflect the model choice based on simulated data from the mean and covariance of

the parameters from the real data. The figure shows predictions from Model 1 and Model 3. The difference between on policy predictions of Model 1 (dark blue) and Model 3 (red) was negligible. A larger difference between the models can be observed for the off policy predictions where Model 1 (cyan) slightly over estimates the first and third reversal. After the initial learning, Model 3 quite consistently underestimates the actual choice probability (89% under estimated trials between trial 25 and 150) and underestimates the second reversal. Grey areas represents reversal onsets. (**b**) The expected value of the two available choices (index finger and middle finger; N = 26 participants). The initial expected value in the model for both choices were set to 0. As seen in Fig 3a. (black line) most individuals (92%) initiated the experiment with the index finger, and since at this point there was a 80% chance of reward, this choice was immediately updated to a higher expected value. At the reversals the expected value of the index finger decrease while the expected value of the middle finger increase over a couple of trials which is congruent with the observed perseverance errors. Note that in the model rewards were coded as 1 and no-reward as -1 which is why the modelled expected value can be negative. Grey areas represents reversal onsets. (**c**) Model recovery confusion matrix (N = 100 simulations per model). Perfect model recovery constitutes an identity matrix, here only Model 1 recovers. Numbers in cells represent percentage best fit. (**d**). Simulating Model 3 with different distributions of $\alpha^+$ and $\alpha^-$ (N = 100 simulations). First column shows the distribution of simulated $\alpha^+$ and $\alpha^-$ generated from the mean and covariance of the real data (same as in Fig. 3c). Model 3 was then simulated and fit with small $\alpha^+$ and large $\alpha^-$ which showed slightly better recovery. Model 3 was then simulated and fit with large $\alpha^+$ and small $\alpha^-$, this combination of parameters recovered. However Model 3 was still not fully distinguishable from Model 1. Boxplots show the median and 25th and 75th percentiles, with the whiskers extending max. 1.5 * interquartile range. (**e**) Simulated adaptive RPE sensitivity for Model 4 and 5 (N = 100 simulations per model). Both Model 4 and 5 used a set of parameters that updated RPE sensitivity for each trial depending on if it encountered rewards or no rewards. Though the models were not recovered (see Fig. 3c) the simulated data generated from the real parameters show that RPE sensitivity was modulated in congruence with the reversals, indicating that the model simulations were successful. The modulation around the reversals were however small. Grey areas represents reversal onsets. Source data are provided as a Source Data file. M1 – Model 1, M3 – Model 3, M4 – Model 4, M5 – Model 5, EV – Expected Value, RPE – Reward Prediction Error.

Note, according to the raw maximum likelihood the winning model included a separate estimation for RPE sensitivity depending on positive or negative outcome (Model 3). However, accounting for extra parameters, using Bayesian Information Criterion (BIC) this model was not better than the simplest model (Model 1). Note that BIC is more conservative than AIC as it punishes more strongly for the number of parameters. As such, AIC seldom finds evidence for the simplest model. The fact that more complex models do not outperform the simplest model in terms of BIC strongly suggests that their complexity is not justified by the data. The only model that could be reasonable excluded was a model that fit separate RPE sensitivities depending on task phase (i.e. separate estimation during stable and volatile periods of the task; Model 2). Model comparison is depicted in Table 1. Choice probability on and off policy for Model 1 and Model 3 is depicted in Fig. 3a. The expected value of the two choices Model 1 is depicted in Fig 3b. A parameter and model recovery analysis was performed using Model 1, 3, 4, and 5. The mean and covariance of the estimated parameters fitted under the real data was used to generate new synthetic parameters which then generated simulated choice data. The simulated data was fitted again with the same model that generated the data and to the other three models. Recovery of the parameters consists of a reasonable correlation between the synthetic parameters and the fitted parameters obtained from the simulated data. N = 100 simulations were generated. All parameters from the different models were reasonable recovered ($r > 0.7$) except the parameter κ used in Model 4 and Model 5 (Table 2). It is likely that our experimental design was not able to disentangle the parameters of Model 4 and making them unsuitable models for this data. Note that since the simulated data was generated using values bound by the estimated parameters the failure of this model is specific to this dataset, it is likely that there exists another combination of parameters where all parameters can be recovered. The model recovery analysis showed that only Model 1 was recovered (83% accuracy). Data generated with the other models favored Model 1, indicating a failure in recovery for Model 3, 4 and 5. We further investigated why these models failed to recover and if the simulations behaved as expected. First we simulated different distributions of $\alpha^+$ and $\alpha^-$ for Model 3. Model 3 only recovered when $\alpha^+$ was large and $\alpha^-$ was small (Fig. 3d). For Model 4 and 5, the failure in recovery likely stems from the parameter κ not generating enough variance in the data. The simulated data for these models behaved as expected, i.e. increasing α (RPE sensitivity) for confirmatory outcomes

and decreasing $\alpha$ for contradictory outcomes (observed as a decrease at the reversal onsets and an increase until the next reversal; Fig. 3e). However, calculating the difference in $\alpha$ one trial before the reversals versus two trials after the reversals showed a small average change (Model 4: M = 0.025, SD = 0.003; Model 5: M = 0.034, SD = 0.003). It is possible that the small changes in $\alpha$ (and the extra parameter needed for the $\alpha$ adaption) causes the failure to recover compared to the model with less parameters. Data and code for the model recovery is available at https://github.com/grillfilip/Dopamine-release-in-human-associative-striatum-during-reversal-learning/tree/main/model_recovery.

## PET simulations and additional basis functions on real data

### *Simulations*

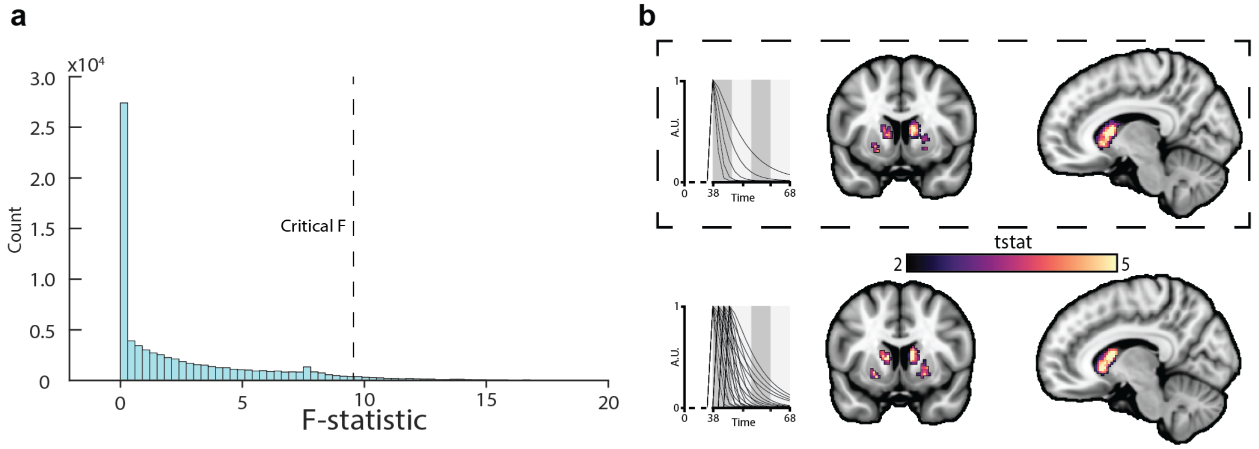


**Fig. 4.**

**Simulated PET data and voxelwise basis functions.** (**a**) TACs were simulated with added noise in the range of 0.1% to 30% which matched the estimated noise from the real data (N = 75500 simulations). The lp-ntPET model was fit to these simulated TACs which was then compared to the MRTM model fit yielding an F-statistic derived from equation 7. F > 9.55 was considered significant (p = 0.05) and since no displacement was added to the simulation any significant fit is a false positive. If the lp-ntPET model over estimates displacement due to noise the number of F-statistics above 9.55 should be more than 5%. The observed false positive rate was 4.86% which indicates that the lp-ntPET model does not over estimate displacement due to noise. The large number of F = 0 means that for those fits MRTM was a better fit than lp-ntPET. (**b**) Voxel wise results on the group level for two different sets of basis functions. The top row shows the results from the basis function set included in the main manuscript and is highly similar to the bottom row (Dice score = 0.85) which includes 5 basis functions at each frame (20 in total) of the PET data during the first reversal. This indicates that the extra set of basis functions did not capture additional displacement compared to the more sparse basis functions (N = 26 participants). Grey areas represents reversal onsets. A.U. – Arbitrary Units.

To better understand potential bias in the modelling of the PET data, TACs were simulated with different levels of noise. First, noise from the real data was estimated using the residuals of each frame from the MRTM fit of the putamen (i.e. an area of the striatum that did not exhibit DA release). The average noise over the group was estimated to 2.93% with a range of 0.06% - 28.75% (three outliers were removed from this estimation). Second, a vector with the length of the TAC of random noise was generated 500 times for each noise level (range from 0.1% to 30%) which then scaled each frame by ± 0.5*noise level (allowing for increases and decreases in the TAC due to the noise). A total of 75500 simulated TACs were generated. The simulated data were then fit with the lp-ntPET model and F-statistics were calculated for each fit compared to the MRTM fit (Normandin et al. 2012):

$$F = \frac{\left(\frac{SSE_{MRTM} - SSE_{lpnt}}{p_{lpnt} - p_{MRTM}}\right)}{\left(\frac{SSE_{lpnt}}{n - p_{lpnt}}\right)} \qquad (7)$$

where SSE is the sum of squared errors for the two models MRTM and lp-ntPET, p is the number of parameters, for MRTM p = 2 and for lp-ntPET p = 3, and n is the total number of frames, in the case of the simulations n = 70. Critical F(2,3) = 9.55 (p = 0.05), the degrees of freedom was taken from $p_{MRTM}$ and $p_{lpnt}$. If the lp-ntPET model overestimates displacement the percentage of fits in this noise range above the critical F value would be expected to be above 5% (i.e. false positives).

The percentage of fits over the critical F value was 4.86% indicating that the model is not over estimating displacement due to noise in the data (Fig. 4a).

To ensure validity of the voxelwise analysis the basis function set was expanded with an additional 15 functions to cover the onset of each frame during the first reversal period. The same analysis procedure was done for this set of basis functions as described in the main manuscript which yields a group parametric map of significant voxels that exhibit displacement. The result showed highly similar voxel statistics to the more sparse set of basis functions used in the main manuscript. The overlap between the two parametric maps showed a Dice score of 0.85 which indicates a substantial correspondence of voxels. This provides evidence that the extra set of basis functions after the reversal did not capture additional displacements of the TACs (Fig. 4b).

***PET model over all reversal events***

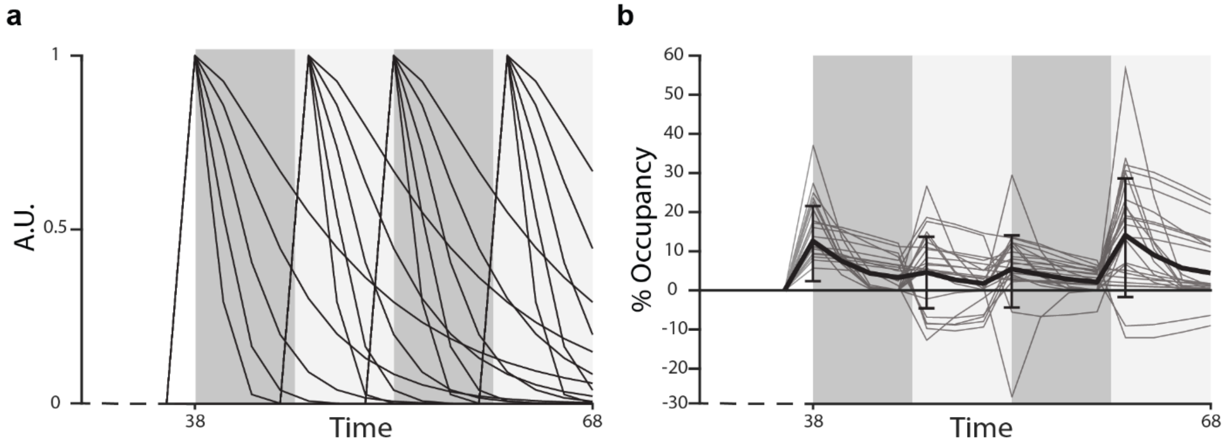


**Fig. 5.**

**Basis functions for each reversal and combined fits.** (**a**) Basis functions covering the reversal events. Grey areas represents reversal onsets (**b**) Using the basis functions covering all reversals, a set of combined functions was fit to the TACs for each participant from the significant DA release ROI. The best combined function fit is displayed for each participant (gray lines). Though the mean fit (black line) over the group looks reasonable, several individuals show negative occupancy at reversal 2, 3, and 4 while all participants show positive occupancy at the first reversal. For these reasons, only the estimation from the first reversal were considered reliable and used for further investigation in the main manuscript. Error bars represent standard deviation of each peak (N = 26 participants). Grey areas represents reversal onsets. Source data are provided as a Source Data file. A.U. – Arbitrary Units.

To investigate DA release for the consecutive reversals, a lp-ntPET model was fit to the TACs of the significant DA release ROI for each participant that included basis functions that covered these events (Fig. 5a). The model seeks to fit the best possible combinations of basis functions to explain the data yielding a continuous estimation of DA occupancy (Fig. 5b). On the mean level over the group, the estimation looks reasonable. However, the individual level functions show negative occupancy values for some participants at the later reversals as well as some participants where a basis function only to the first reversal best explained the data. For these reasons, only the first reversal event was considered to yield a reliable estimation and was used for the results in the main manuscript. As discussed in the main text, this might reflect a specific cognitive process occurring at the first reversal, but could also, at least in part, be due to the lower radioactivity and slow pharmacokinetics of [11C]Raclopride during these later time points.

***Single subject data and occupancy association with confounding variables***

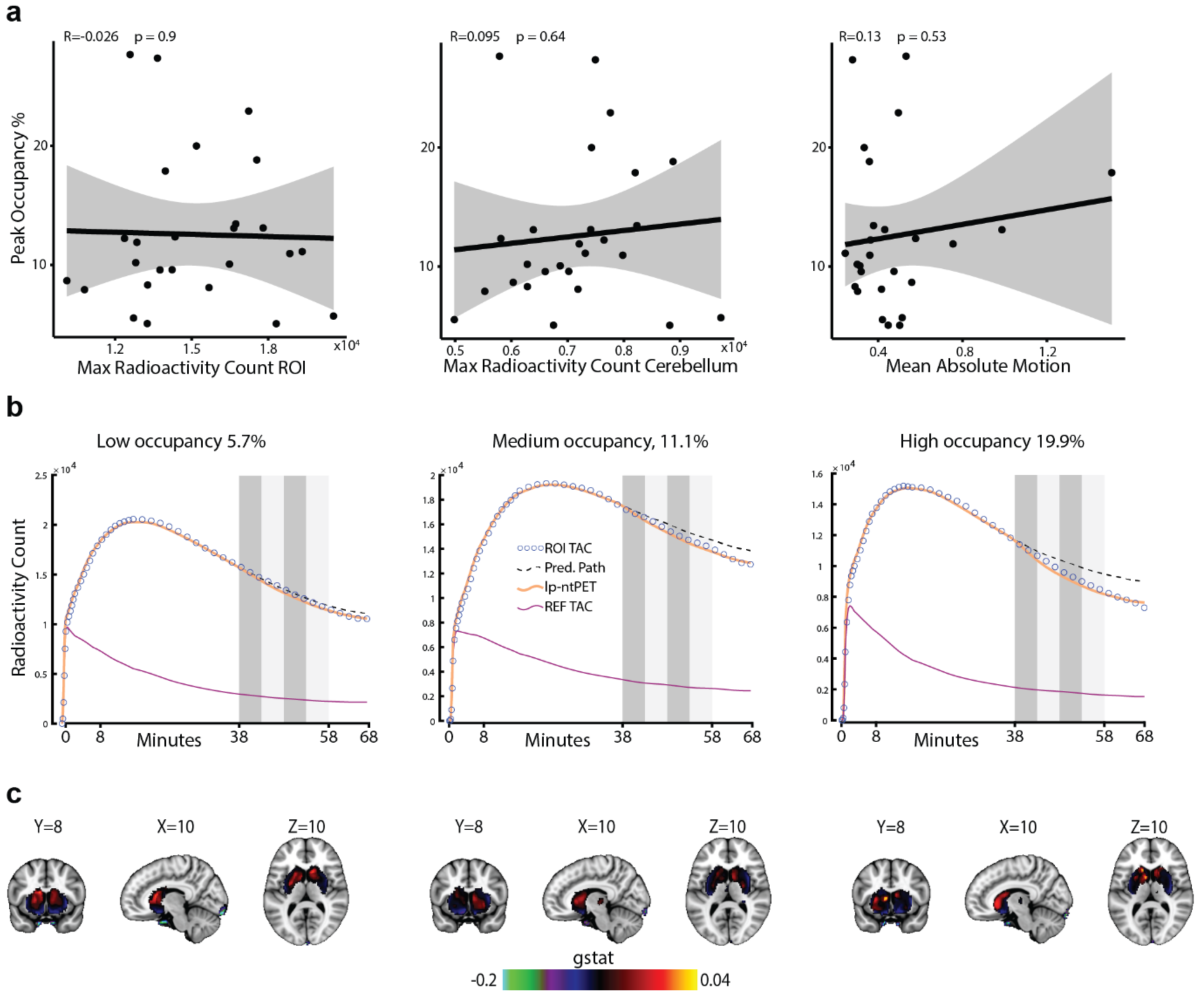


**Fig. 6.**

**Confound associations and single subject data.** (**a**) Confound Pearson correlations (N = 26 participants) between peak occupancy and maximum radioactivity count in the ROI TAC, maximum radioactivity count in the reference TAC (cerebellum), and the mean absolute motion of each participant. No significant association was observed. **S**haded areas represent 95% confidence interval**.** (**b**) Single subject PET data for three representative subjects with different magnitudes of occupancy. As in Fig. 2C, TAC from the significant cluster voxelwise group cluster, lp-ntPET fit, predicted path (i.e. the path of the TAC if no DA was released), and reference TAC (cerebellum) for the individuals are depicted. Grey areas represents reversal onsets. (**c**) Voxelwise parametric maps of the scaling (Gstat) of the best fitted function for the same individuals as in b. Source data are provided as a Source Data file. ROI – Region of Interest, TAC – Time Activity Curve, Pred. – Predicted, lp-ntPET – linear parametric neurotransmitter Positron Emission Tomography, REF – Reference.

Single subject data for representative participants with low, medium, and high dopamine occupancy is shown in Fig. 6a. The data was extracted from the group level significant cluster and shows the different TAC profiles and model fits. The corresponding voxelwise maps for these subjects are shown in Fig. 6b. Note that both positive and negative parameters are present in the maps since the model is allowed to fit both positive and negative displacement to not bias the results towards positive displacement. To further investigate model bias, occupancy was correlated with potential confounding variables: maximum radioactivity count in the ROI TAC of interest, maximum radioactivity count in the reference TAC, and the mean absolute motion of each participant (Fig. 6c). No association between occupancy and confounds was observed using Bayes factor correlation (null hypothesis tested against an alternative hypothesis of a medium correlation of r = 0.33). Bayes

factor for ROI TAC was 0.43, REF TAC was 0.46, and motion was 0.50, indicating in favor for the null hypothesis.

## fMRI model information

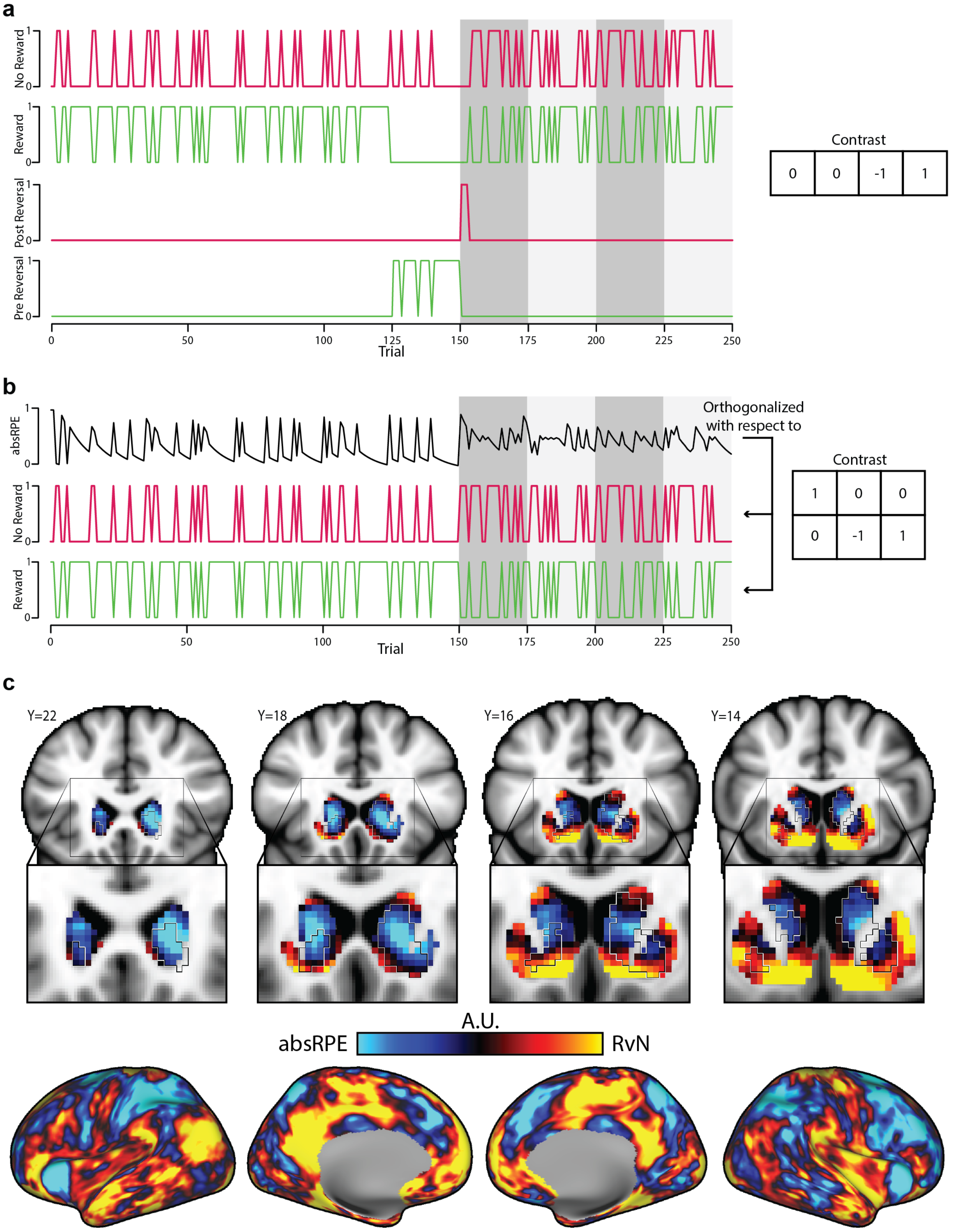


**Fig. 7.**

**First level models and group visualization.** (**a**) An example of what the perseverance error fMRI model might look like for a single participant (simulated data). Four regressors were entered into the model: non-rewarded outcomes (No Reward), rewarded outcomes (Reward), non-rewarded outcomes post the first reversal until a rewarded outcome that was elicited by a correct response (i.e. choice reversal taken place; Post Reversal), and rewarded correct

responses 25 trials before the first reversal (Pre Reversal). The contrast of interest was Post Reversal > Pre Reversal (i.e. BOLD signal related to the same choice but different outcome. Grey areas represents reversal onsets. (**b**) An example of what the absRPE fMRI model might look like for a single participant (same as in Fig 7a). absRPE was extracted from the computational behavioral Model 1 and entered as a covariate to a model that included non-rewarded and rewarded outcomes. As can be seen, absRPE is sometimes colinear with the other regressors. absRPE was therefore orthogonalized with respect to the other two regressors. This assigns the shared variability for both Reward and No Reward regressors to the absRPE covariate. As a control contrast, Reward>No Reward was used to confirm that a canonical reward BOLD response was observed after the orthogonalization. Note that this model includes all components of a signed RPE, i.e. valence and surprise, but is able to isolate the surprise component trough absRPE. (**c**) For visualization purposes the contrast Reward>No Reward (RvN, yellow) was contrasted against the covariate of absolute reward prediction error (RPE, blue) on the group level to show voxels that more strongly represent one or the other (N = 26 participants). The outline in the striatum represents the significant DA release cluster. A canonical reward response can be seen in the ventral striatum; dorsal to the reward response, RPEs are more strongly represented which show more pronounced overlap with the DA release cluster compared to the reward response. Cortically, RPEs were more represented in the fronto-parietal and ventral attention networks including the insula, DLPFC, parietal cortex, and anterior cingulate cortex while a reward response was seen in the limbic and default mode networks including the ventromedial prefrontal cortex and precuneus. Note that this contrast was not a statistical test, statistics are therefore not reported. MNI-coordinates. absRPE – absolute Reward Prediction Error, RvN – Reward versus No reward, A.U. – arbitrary units.

A visualization of the perseverance error fMRI model is depicted in Fig. 7a. A visualization of the absRPE fMRI model is depicted in Fig. 7b. The main manuscript, (see Main Manuscript; Fig 3b) shows the significant BOLD response to the absRPE covariate. For completeness and visualization purposes the valence regressor contrast (Reward > No Reward) was contrasted against the RPE covariate to show which voxels displayed mostly represented the valence contrast compared to the covariate (Fig. 7c).

**BOLD signal association with DA occupancy**

Using the significant DA release cluster, individual absRPE beta estimates were extracted. The absRPE betas as well as its quadratic term was used as predictors to explain peak DA occupancy. No significant associations were found (Fig 8a). A similar analysis was performed whole brain where peak DA occupancy and its quadratic term was entered as covariates to explain absRPE beta estimates. Fig. 8b shows the linear association. No significant voxels were observed. Fig 8c. shows the quadratic association. No significant voxels were observed.

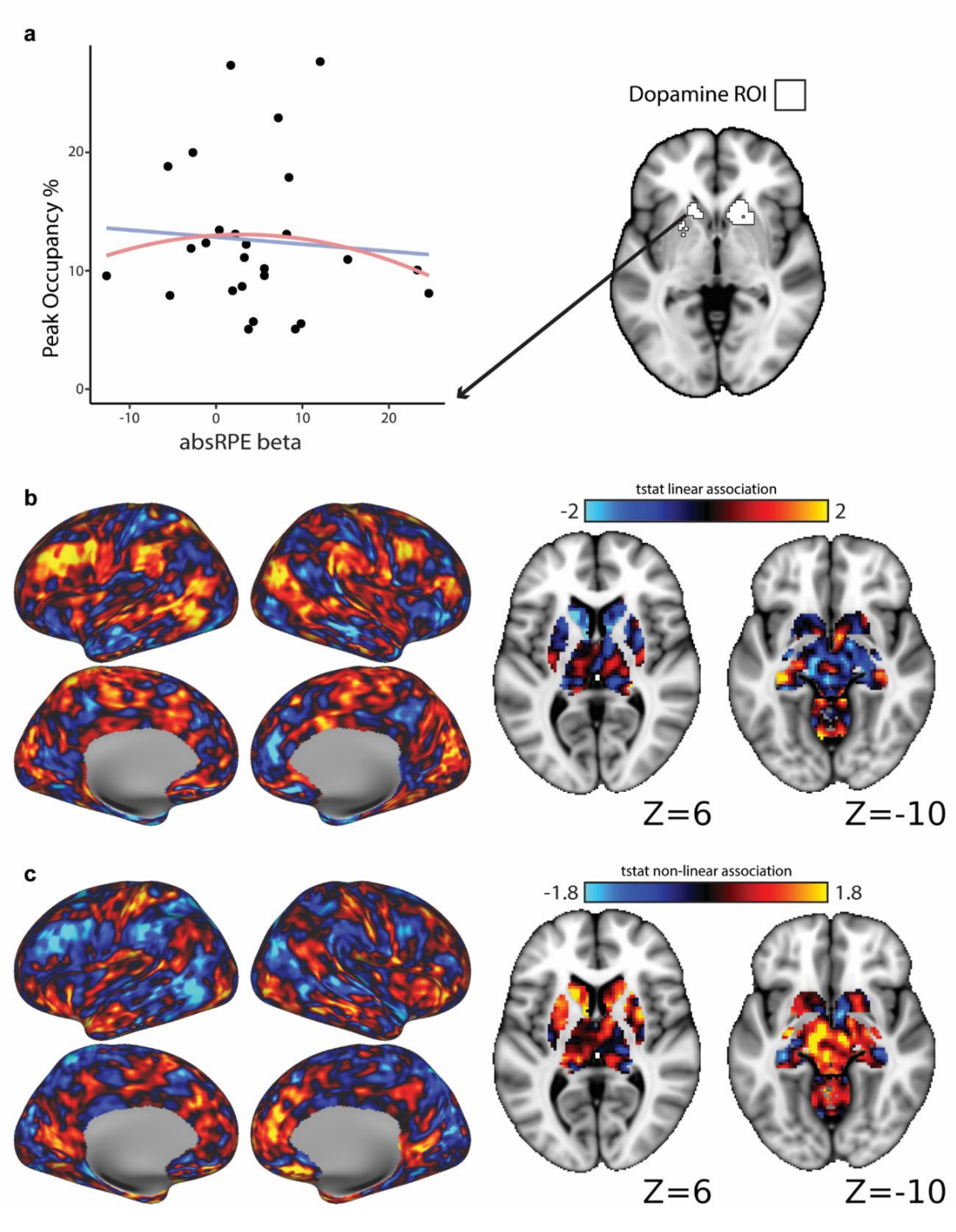


**Fig. 8.**

**absRPE BOLD association with DA occupancy.** (**a**) Using the significant dopamine release cluster as a ROI, the absRPE beta estimates were extracted and entered as linear and quadratic predictors to explain peak DA occupancy.

No significant associations were observed (N = 26 participants; absRPE: t = 0.18, p = 0.86; absRPE$^2$: t = 0.56, p = 0.56). (**b**) Exploratory whole brain linear association between peak DA occupancy and absRPE. No significant voxels were observed (N = 26 participants). (**c**) Exploratory whole brain quadratic association between peak DA occupancy and absRPE. No significant voxels were observed (N = 26 participants). Source data are provided as a Source Data file. ROI – Region of Interest, absRPE – Absolute Reward Prediction Error.

## Supplementary References


1. Rescorla, R. A. & Wagner, A. R. A theory of Pavlovian conditioning and the effectiveness of reinforcement and non-reinforcement. In: Classical conditioning. 2. Current research and theory. (1972).
2. Sutton, S. R. & Barto, G. A. *An introduction to reinforcement learning*. *MIT press* (1998). doi:10.4018/978-1-60960-165-2.ch004.
3. Frank, M. J., Moustafa, A. A., Haughey, H. M., Curran, T. & Hutchison, K. E. Genetic triple dissociation reveals multiple roles for dopamine in reinforcement learning. *Proc. Natl. Acad. Sci. U. S. A.* **104**, 16311–16316 (2007).
4. Niv, Y., Edlund, J. A., Dayan, P. & O'Doherty, J. P. Neural prediction errors reveal a risk-sensitive reinforcement-learning process in the human brain. *J. Neurosci.* **32**, 551–562 (2012).
5. Cazé, R. D. & Van Der Meer, M. A. A. Adaptive properties of differential learning rates for positive and negative outcomes. *Biol. Cybern.* **107**, 711–719 (2013).
6. Gershman, S. J. Do learning rates adapt to the distribution of rewards? *Psychon. Bull. Rev.* **22**, 1320–1327 (2015).
7. Behrens, T. E. J., Woolrich, M. W., Walton, M. E. & Rushworth, M. F. S. Learning the value of information in an uncertain world. *Nat. Neurosci.* **10**, 1214–1221 (2007).
8. Vinckier, F. *et al.* Confidence and psychosis: A neuro-computational account of contingency learning disruption by NMDA blockade. *Mol. Psychiatry* **21**, 946–955 (2016).
