# Supplementary material for: Dopamine release in human associative striatum during reversal learning
Source: Nat Commun. 2024 Jan 2;15:59. doi: 10.1038/s41467-023-44358-w (PMC10762220; doi:10.1038/s41467-023-44358-w)
Supplement: Supplementary file 3 — Reporting Summary [file 41467_2023_44358_MOESM3_ESM.pdf]

nature portfolio

Corresponding author(s): Filip Grill
Anna Rieckmann

Last updated by author(s): Oct 8, 2023

Reporting Summary

Nature Portfolio wishes to improve the reproducibility of the work that we publish. This form provides structure for consistency and transparency in reporting. For further information on Nature Portfolio policies, see our Editorial Policies and the Editorial Policy Checklist.

Statistics

For all statistical analyses, confirm that the following items are present in the figure legend, table legend, main text, or Methods section.

n/a	Confirmed	
☐	☒	The exact sample size (*n*) for each experimental group/condition, given as a discrete number and unit of measurement
☐	☒	A statement on whether measurements were taken from distinct samples or whether the same sample was measured repeatedly
☐	☒	The statistical test(s) used AND whether they are one- or two-sided<br>*Only common tests should be described solely by name; describe more complex techniques in the Methods section.*
☐	☒	A description of all covariates tested
☐	☒	A description of any assumptions or corrections, such as tests of normality and adjustment for multiple comparisons
☐	☒	A full description of the statistical parameters including central tendency (e.g. means) or other basic estimates (e.g. regression coefficient) AND variation (e.g. standard deviation) or associated estimates of uncertainty (e.g. confidence intervals)
☐	☒	For null hypothesis testing, the test statistic (e.g. *F*, *t*, *r*) with confidence intervals, effect sizes, degrees of freedom and *P* value noted<br>*Give P values as exact values whenever suitable.*
☐	☒	For Bayesian analysis, information on the choice of priors and Markov chain Monte Carlo settings
☐	☒	For hierarchical and complex designs, identification of the appropriate level for tests and full reporting of outcomes
☐	☒	Estimates of effect sizes (e.g. Cohen's *d*, Pearson's *r*), indicating how they were calculated

Our web collection on statistics for biologists contains articles on many of the points above.

Software and code

Policy information about availability of computer code

Data collection	GE Signa PET/MR, PsychoPy2 (1.85.2)
Data analysis	Code and data available at https://zenodo.org/records/10100769 (DOI: 10.5281/zenodo.10100769)<br>Software: MATLAB (R2017b), R (4.0.2), rSTAN (2.26.1), FSL (5.0), FreeSurfer (6.0), Workbench Command (1.3.2)

For manuscripts utilizing custom algorithms or software that are central to the research but not yet described in published literature, software must be made available to editors and reviewers. We strongly encourage code deposition in a community repository (e.g. GitHub). See the Nature Portfolio guidelines for submitting code & software for further information.

Data

Policy information about availability of data

All manuscripts must include a data availability statement. This statement should provide the following information, where applicable:

- Accession codes, unique identifiers, or web links for publicly available datasets
- A description of any restrictions on data availability
- For clinical datasets or third party data, please ensure that the statement adheres to our policy

The group- and individual level processed brain imaging data are available at https://zenodo.org/records/10100769 (DOI: 10.5281/zenodo.10100769). The behavioral data generated in this study are provided in the Source Data file. The unprocessed research data is available upon request from qualified researchers, provided that ethical and legal restrictions that govern data sharing are met. Participants in this study did not provide informed consent for public data sharing.

Requests for data access should be directed to anna.rieckmann@unibw.de and will be dealt with promptly.

Research involving human participants, their data, or biological material

Policy information about studies with human participants or human data. See also policy information about sex, gender (identity/presentation), and sexual orientation and race, ethnicity and racism.

Reporting on sex and gender	The effect of sex or gender was not considered in the current study due to lack of hypotheses regarding sex/gender effects. Participants gender identity was determined based on self-reporting. Consent for openly sharing individual-level data has not been obtained.
Reporting on race, ethnicity, or other socially relevant groupings	Data regarding race, ethnicity, or other socially relevant groupings were not collected at the time of data collection and is therefore not reported.
Population characteristics	Exclusion criteria consisted of current or past diagnosis of neurological or psychiatric illness, claustrophobia, history of head trauma, alcohol or drug dependence, and use of psychopharmaceuticals, drugs, or stimulants other than caffeine or nicotine for the past 6 months. Individuals with MRI-incompatible implants or objects were excluded for MRI safety reasons. Individuals that had previously undergone PET scanning for research purposes as well as pregnant or breast-feeding individuals were excluded for radiation safety reasons. Final sample consisted of twenty-six participants (13 female; mean age = 25.73; SD = 4.57; range = 20 – 36).
Recruitment	Participants were recruited through postings on public bulletin boards around Umeå University as well as through opt-in social media groups related to research projects at Umeå University. A majority of participants were university students which is not likely to be representative of the population as a whole.
Ethics oversight	This study was approved by the Regional Ethics Committee at Umeå University (2015/239-31).

Note that full information on the approval of the study protocol must also be provided in the manuscript.

Field-specific reporting

Please select the one below that is the best fit for your research. If you are not sure, read the appropriate sections before making your selection.

☐ Life sciences ☒ Behavioural & social sciences ☐ Ecological, evolutionary & environmental sciences

For a reference copy of the document with all sections, see nature.com/documents/nr-reporting-summary-flat.pdf

Behavioural & social sciences study design

All studies must disclose on these points even when the disclosure is negative.

Study description	The study contains quantitative experimental data.
Research sample	Representative sample of 30 healthy young adult participants, 50% female, 50% male. Age: range 20 to 36, mean 25.73, standard deviation 4.57 years. Sample was chosen to investigate effects in healthy young adults with a putatively mature and intact dopamine signaling.
Sampling strategy	Sampling was made based on age criteria (18 to 40) and to, as far as possible, balance males and females. The sample size was chosen based on an a priori power analysis with an effect size of r = 0.5 with 80% power giving a required sample size of 30. Random sampling strategy was used.
Data collection	Stimuli was presented on a BOLDscreen 23" LCD for fMRI (Cambridge Research Systems) connected to a MacBook Air (2017) running PsychoPy2 (1.85.2). Responses were recorded through a Pyka hand held response pad connected to a FIU-905 interface (which was connected to the MacBook Air). MRI and PET data were simultaneously collected with a GE Signa PET/MR. Participants were alone in the scanner room. Researchers were not blind to the experimental condition and study hypothesis during data collection.
Timing	March 2020 - December 2020
Data exclusions	Four participants were excluded from the study due to technical reasons relating to the timing of the PET/fMRI acquisition.
Non-participation	No participants dropped out/declined participation.
Randomization	Participants were not allocated into experimental groups. Due to the tight age range and no a priori assumption on effects of sex/gender, these covariates were not considered in the analysis.

Reporting for specific materials, systems and methods

We require information from authors about some types of materials, experimental systems and methods used in many studies. Here, indicate whether each material, system or method listed is relevant to your study. If you are not sure if a list item applies to your research, read the appropriate section before selecting a response.

Materials & experimental systems

n/a	Involved in the study
☒	☐ Antibodies
☒	☐ Eukaryotic cell lines
☒	☐ Palaeontology and archaeology
☒	☐ Animals and other organisms
☒	☐ Clinical data
☒	☐ Dual use research of concern
☒	☐ Plants

Methods

n/a	Involved in the study
☒	☐ ChIP-seq
☒	☐ Flow cytometry
☐	☒ MRI-based neuroimaging

Plants

Seed stocks	*Report on the source of all seed stocks or other plant material used. If applicable, state the seed stock centre and catalogue number. If plant specimens were collected from the field, describe the collection location, date and sampling procedures.*
Novel plant genotypes	*Describe the methods by which all novel plant genotypes were produced. This includes those generated by transgenic approaches, gene editing, chemical/radiation-based mutagenesis and hybridization. For transgenic lines, describe the transformation method, the number of independent lines analyzed and the generation upon which experiments were performed. For gene-edited lines, describe the editor used, the endogenous sequence targeted for editing, the targeting guide RNA sequence (if applicable) and how the editor was applied.*
Authentication	*Describe any authentication procedures for each seed stock used or novel genotype generated. Describe any experiments used to assess the effect of a mutation and, where applicable, how potential secondary effects (e.g. second site T-DNA insertions, mosiacism, off-target gene editing) were examined.*

Magnetic resonance imaging

Experimental design

Design type	Task; event-related.
Design specifications	Participants completed 250 trials. Each trial lasted 6 seconds. Between each trial there was a inter trial interval with jittered duration of 1 to 13 seconds. The inter trial interval was drawn from a distribution and designed so that 25 trials always took 5 minutes to complete.
Behavioral performance measures	The first button press (two possible) of each trial within the response window was recorded and indicated the choice participants made. Performance was of interest in the study and relates to the total amount of win trials. The number of missed trials were checked for each participant.

Acquisition

Imaging type(s)	Functional, structural
Field strength	3T
Sequence & imaging parameters	The BOLD fMRI data was acquired for 50 minutes with the following parameters: FOV: 25.6, Matrix: 96 × 96, Slice Thickness: 3.6 mm, TE: 30 ms, TR: 4,000 ms, Flip Angle: 90°, Acceleration Factor: 2.0, resulting in a voxel size of 1.95 x 1.95 x 3.9 mm3.<br><br>Structural T1-weighted images were acquired for 7.36 min with the following acquisition parameters: [FOV: 25 × 20 cm2, matrix: 256 × 256, Slice Thickness: 1 mm, Slices: 180, TE: 3.1 ms, TR: 7,200 ms, Flip Angle: 12, Bandwidth: 244.1 Hz/Pixel].
Area of acquisition	Whole brain

Diffusion MRI ☐ Used ☒ Not used

Preprocessing

Preprocessing software	FSL FEAT (FMRI Expert Analysis Tool) 6.00. Data were smoothed with a Gaussian kernel (FWHM = 8 mm)
Normalization	The functional data was linearly normalized to the structural image. The structural image was first linearly registered to a template image then further refined through non-linear registration. The resulting linear matrix and displacement field was then used to warp the functional data to the standard template.

Normalization template	MNI152 2mm
Noise and artifact removal	6 motion parameters were included in all GLM analyses.
Volume censoring	No volume censoring was performed

Statistical modeling & inference

Model type and settings	Mass univariate GLM. Two models were defined for the data at the first level (1) perseverance errors model: (a) regressor containing correct response trials inside 25 trials of the first reversal, (b) regressor containing trials of incorrect response trials (i.e. continuing with the previously correct response until switching to the current correct response) after the first reversal, (c) regressor containing all other trials as regressor of no interest. (2) RPE covariate model: (a) regressor with all rewarding trials of no interest, (b) regressor with all non-rewarding trials of no interest, (c) trial-by-trial covariate of reward prediction errors defined from the computational cognitive model. Second level group results were obtained using random effects permutation testing.
Effect(s) tested	Contrast of interest were [incorrect response trials after the first reversal > correct response trials inside 25 trials of the first reversal] and the effect of the RPE covariate. Effects tested using one-sample t-test (one-sided).

Specify type of analysis: ☐ Whole brain ☐ ROI-based ☒ Both

Anatomical location(s): Reversal contrast investigated in a striatal ROI defined from the PET analysis.

Statistic type for inference (See Eklund et al. 2016)	Voxel-wise k > 10 voxels using perumtation testing
Correction	Threshold free cluster enhancement (TFCE), small volume correction for the reversal contrast and whole brain correction for the RPE covariate.

Models & analysis

n/a	Involved in the study
☐	☒ Functional and/or effective connectivity
☒	☐ Graph analysis
☒	☐ Multivariate modeling or predictive analysis

Functional and/or effective connectivity: Pearson correlation
